# A Novel Antibacterial Titanium Modification with a Sustained Release of Pac-525

**DOI:** 10.3390/nano11123306

**Published:** 2021-12-06

**Authors:** Yuzhu He, Yuanyuan Li, Enjun Zuo, Songling Chai, Xiang Ren, Tao Fei, Guowu Ma, Xiumei Wang, Huiying Liu

**Affiliations:** 1 School of Stomatology, Dalian Medical University, Dalian 116044, China; yuzhuh0723@dmu.edu.cn (Y.H.); yuanyuan0154@163.com (Y.L.); zuoej@163.com (E.Z.); chaisongling@hotmail.com (S.C.); renxiangdy@foxmail.com (X.R.); dyfeitao@sina.cn (T.F.); 2 State Key Laboratory of New Ceramics and Fine Processing, School of Materials Science and Engineering, Tsinghua University, Beijing 100084, China

**Keywords:** titanium modification, electrospray, acid–alkali heat pretreated mineralization, Pac-525

## Abstract

For the benefit of antibacterial Ti on orthopedic and dental implants, a bioactive coating (Pac@PLGA MS/HA coated Ti) was deposited on the surface of pure titanium (Ti), which included two layers: an acid–alkali heat pretreated biomimetic mineralization layer and an electrosprayed Poly (D,L-lactide-co- glycolic acid) (PLGA) microsphere layer as a sustained-release system. Hydroxyapatite (HA) in mineralization layer was primarily prepared on the Ti followed by the antibacterial coating of Pac-525 loaded by PLGA microspheres. After observing the antimicrobial peptides distributed uniformly on the titanium surface, the release assay showed that the release of Pac-525 from Pac@PLGA MS/HA coated Ti provided a large initial burst followed by a slow release at a flat rate. Pac@PLGA MS/HA coated Ti exhibited a strong cytotoxicity to both Gram-negative bacteria (*Escherichia coli*) and Gram-positive bacteria (*Staphylococcus aureus*). In addition, Pac@PLGA MS/HA coated Ti did not affect the growth and adhesion of the osteoblast-like cell line, MC3T3-E1. These data suggested that a bionic mineralized composite coating with long-term antimicrobial activity was successfully prepared.

## 1. Introduction

Orthopedic implants and dental implants are widely used to restore missing bones or teeth. Most orthopedic implants and dental implants are made of titanium (Ti) and its alloys as they show good biocompatibility, low modulus of elasticity, and high resistance to corrosion [1,2]. The success of osseointegration between new bone and implant is always determined by the features of the materials and the environment of the bone [3]. As the main inorganic component of vertebrate bone (69 wt%) and teeth (96 wt%), hydroxyapatite (HA) was coated on the Ti surface by biomimetic mineralization after acid–alkali heat treatment to promote new bone formation enhancing implant stability through improving the osseointegration [4,5,6]. It is believed that the HA-coating Ti and its alloy would lead to a shorter rehabilitation after surgery [4,7,8].

However, the various etiologies of regeneration failure related to the use of pure Ti and modified Ti have been reported [9,10,11], the reason of which was that exposure to air leads to the formation of an oxide layer, and the lack of antimicrobial properties makes Ti orthopedic implants and dental implants susceptible to bacterial plaques, which will further induce inflammatory reactions [3]. To address this problem, we proposed to improve the antimicrobial ability of Ti by surface modification. The surface modification of the Ti implant is a probable way to avoid the occurrence of implant-related infection, which can be achieved through two main categories: modifying the surface directly and coating on the surface of Ti [12,13]. Although modifying the Ti surface simply with a physical and chemical technique is a promising approach, some difficulties remain to be overcome, such as the stability of the immobilized biomolecules, the release time of the physically adsorbed agent, and the risk of reducing bioactivity during the use of the chemical reaction [14]. For example, the antibacterial agent loaded into the calcium phosphate coating on Ti directly was found to perform a completely burst release from the coating within the first 60 min [15]. This consequence indicated that using HA coating desorption directly was not an advisable strategy for loading antibacterial agents. PLGA (Poly (D,L-lactide-co-glycolic acid)) has excellent performance on biodegradability, biocompatibility, and antigenicity [16,17], the negative charge on the surface of which (carboxyl terminal) could influence its interaction with bacteria [18,19]. PLGA microspheres (PLGA MS) were prepared for avoiding prematurely metabolism and maintaining drug concentration locally through electrospraying technique that displayed benefits on reducing residual organic solvents and affording tighter regulation over particle size distribution and morphology [20]. Ideally, the microbial was prevented from adhering on the implant due to the existence of the PLGA microsphere layer, which also released the antibacterial agent as a drug delivery system.

Compared to the traditional antibiotics, antimicrobial peptides (AMPs) are a tempting option, owing to their advantage of high efficacy on broad-spectrum antimicrobial activity and low propensity for developing resistance [21,22,23]. In our previous studies, Pac-525 (Pac, Ac-KWRRWVRWI- NH2) [24] as an artificial AMP displayed excellent antibacterial property on inhibiting *Streptococcus sanguis, Staphylococcus aureus, Fusobacterium nucleatum, Porphyromonas gingivalis,* and *Escherichia coli* [25,26]. On based of conventional charge adsorption theory, the rich hydrophobic amino acids (Val, Ile, and Trp in Pac-525) could help it insert deeply into the lipid bilayer of bacteria. Furthermore, Lys and Arg in Pac-525 were also inferred to affect the neutralizing LPS due to its close-packing with the phosphate groups or saccharides of LPS. Therefore, we believe that if Pac-525 were attached to the Ti surface, it would effectively inhibit the occurrence of peri-implant inflammation. On the basis of this observation, it was expected that the existence of Pac@PLGA microspheres would increase the treatment efficiency of an HA-coated Ti implant.

As the current study verified the abovementioned scientific hypothesis, the biomimetic mineralized Ti coating with an antimicrobial peptide Pac-525, Pac@PLGA MS/HA coated Ti, was prepared. The physical properties, biocompatibility, and antibacterial activity of Pac@PLGA MS/HA coated Ti showed its potentiality on improving the antibacterial and biological activity of Ti orthopedic implants and dental implants.

## 2. Materials and Methods

### 2.1. Preparation of Pac@PLGA MS/HA Coated Ti

Pure Ti (10 mm in diameter and 2 mm in thickness) was polished with sand paper and then ultrasonic cleaned with acetone, ethanol, and deionized water for 10 min. Ti was soaked in a mixed solution including HCL (Chemical Reagent Co., Ltd. Beijing, China), H_2_SO_4_ (Chemical Reagent Co., Ltd. Beijing, China), and H_2_O (1:1:2) for 30 min and cleaned ultrasonically with deionized water 3 times for 15 min each time. The Ti was then reacted with 1 M NaOH (Chemical Reagent Co., Ltd. Beijing, China) at 160 °C for 6 h in a reactor and washed 3 times with deionized water for 15 min each time at room temperature. After acid–alkali heat treatment, the Ti was put into DPBS (Dulbecco’s phosphate-buffered saline (GIBCO Invitrogen Corporation/Life Technologies Life Sciences, Carlsbad, CA, USA) solution containing 100 mg/L anhydrous calcium chloride (CaCl_2_) (Chemical Reagent Co., Ltd., Beijing, China) for 24 h at 37 °C to form a biomimetic mineralization layer; 50 μL of Pac-525 (Qiangyao Bio-Technology Co., Ltd. Shanghai, China) solution (10 mg/mL) was dripped onto the surface of HA coated Ti and dried in air.

Then, 200 mg/mL Pac-525 solution was mixed into a 6% PLGA (Medical Equipment Research Institute, Jinan, China) solution. The emulsion was formed by an ultrasonic crasher (Scientz-IID, Ningbo Science Biotechnology Co., Ltd., Ningbo, China) at 300–400 W on ice for 20 s and then added into the 1 mL syringe. The syringe was fixed on the injection pump, and Ti samples were placed on the receiving plate (the distance of reception was 20 cm). The positive voltage for electrospraying was 5 kV and the negative voltage for receiving was 1 kV [20]. PLGA microspheres were sprayed onto the surface of the Ti samples.

### 2.2. Phase Composition Analysis

X-ray diffraction (XRD) was used to analyze the phases of the Ti surface with an X-ray diffractometer (Rigaku Dmax2500, Tokyo, Japan). The following parameters of Cu-Ka radiation were set: wavelength λ = 0.154 nm, step width = 0.02, scanning speed = 4°/min, scanning angles: 20~70°.

### 2.3. Morphology Analysis

The morphology of the coated Ti samples were observed through a scanning electron microscope (SEM, JSM-7001F, Tokyo, Japan) [27]. For confocal microscopy assay, the Pac@PLGA MS is specially grafted with FITC. The biomimetic mineralized components were marked with Rhodamine B. The distribution of Pac-525 in the coating was observed by a confocal laser scanning microscope (CLSM-710-3channel, Zeiss, Oberkochen, Germany

### 2.4. Release Curve Analysis

To measure the total content of Pac-525 in the PLGA MS/HA coating, Pac@PLGA MS/HA coated Ti was soaked in acetonitrile followed by adding 0.1 M HCL. After centrifuging at 5000 rpm/min for 15 min, the concentration of Pac-525 in the supernatant was measured through a microplate reader. The measured concentration of Pac-525 was used to calculate the quality of Pac-525 (M). To investigate the release of Pac-525, the samples were soaked into 1 mL (*V***_0_**) PBS on a shaker (60 rpm/min) at 37 °C. At different time points (*n* = 1, 2, 3, 4, 5, 6, and 7, representing 3, 6, 12, 18, 24, 72, and 120 h), 0.5 mL (*Ve*) PBS was replaced by fresh PBS. The content of Pac-525 at each time point in the released solution was tested using a microplate reader (Perkin Elmer EnSpire, Waltham, MA, USA) [20].

$$En=\frac{V_{e}\times \sum_{1}^{n-1}\times C_{n-1}+V_{0}\times C_{n}}{M}\times 100\%$$

### 2.5. The Biocompatibility of Pac-525 and Pac@PLGA MS/HA Coated Ti

#### 2.5.1. Cell Viability

MC3T3-E1 cells were cultured in an α-MEM medium (GIBCO Invitrogen Corporation/Life Technologies Life Sciences, Carlsbad, CA, USA) containing 10% FBS (fetal bovine serum) (GIBCO Invitrogen Corporation/Life Technologies Life Sciences, Carlsbad, CA, USA) and 1% PS (penicillin–streptomycin) at 37 °C in a humid atmosphere of 5% CO_2_. MC3T3-E1 cells were seeded in a 48-well plate containing Pac-525 (0, 10, 50, 100, 150, and 200 μg/mL) at the density of 2 × 10^4^ cells/well. Cell Counting Kit-8 (CCK-8) (Beyotime Institute of Biotechnology, Jiangsu, China) was used to evaluate the cytotoxicity of Pac-525 at different doses; 0.1 mL of CCK-8 working solution was used to incubate the cells for 3 h. The OD value was measured at 450 nm via a microplate reader [26].

#### 2.5.2. Cell Adhesion and Proliferation

The cells were cultured on Pac@PLGA MS/HA coated Ti for 6 h. The samples were then observed through SEM. Part of the samples were stained with DAPI (Sigma-Aldrich Co., LLC., Rockville, MD, USA) and phalloidin-TRITC conjugate (Invitrogen, CA, USA) and detected with a confocal microscope.

The cytotoxicity of Pac@PLGA MS/HA coated Ti was measured with a Cell Counting Kit-8 (CCK-8). The MC-3T3 cells were cultured on Ti samples for 1, 3, and 5 days after treating the cells with 0.1 mL CCK-8 working solution for 3 h. The OD value was measured at 450 nm via a microplate reader.

### 2.6. Antibacterial Activity of Pac@PLGA MS/HA Coated Ti

#### 2.6.1. Antibacterial Activity Test of Pac-525

The antibacterial activity of Pac-525 was determined by observing the bacterial growth area on the agar plates. One milliliter of *Escherichia coli* (*E. coli*, G-, Gram-negative bacteria) and *Staphylococcus aureus* (*S. aureus*, G+, Gram-positive bacteria) at the dose of 1 × 10^6^ CFU/mL was mixed with 1 mL Pac-525 solution (0, 10, 50, 100, 150, and 200 μg/mL). The mixture was then uniformly spread on LB agar plates. After 8 h incubation, the area of bacteria on the plate was used to reflect the antibacterial activity of Pac-525.

#### 2.6.2. Antibacterial Function of Pac@PLGA MS/HA Coated Ti

The observation on the existence of a bacteriostatic ring on agar plates was recruited to determine the antibacterial property of Pac@PLGA MS/HA coated Ti. The diameter of bacteriostatic ring was measured to reflect the activity of samples. *E. coli* and *S. aureus*, 1 × 10^6^ CFU/mL, was seeded on LB agar plates. Then the Ti samples (with or without Pac-525 @PLGA MS) were placed on the surface of *E. coli*/*S. aureus* agar plates. The formation of bacteriostatic rings was observed 24 h later. For bacteria adhesion analysis, 1 × 10^6^ CFU/mL of *E. coli* and *S. aureus* were seeded on Ti samples in 48-well plates. After 24 h incubation, the bacteria on the Ti samples were observed with SEM.

## 3. Results

### 3.1. Characterization of Pac@PLGA MS/HA Coated Ti

**Figure 1:**
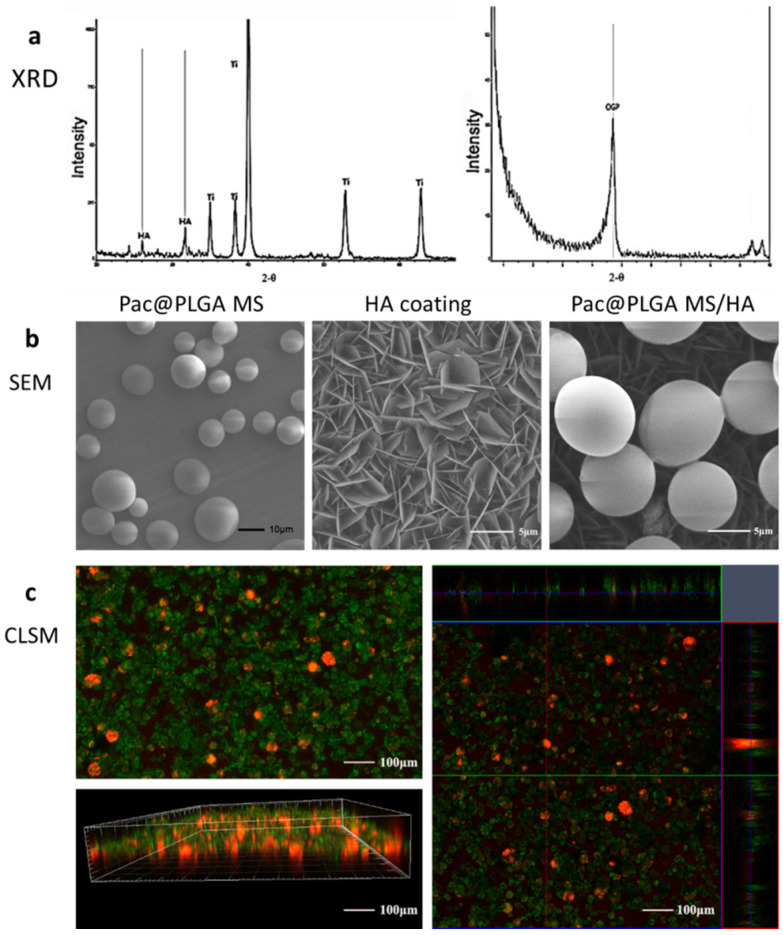
The morphology of PLGA microspheres and HA coating were observed through SEM and CLSM. (**a**) Phase composition of Pac@PLGA MS/HA coating. (**b**) SEM images for the morphology of Pac@PLGA microspheres, HA coating, and Pac@PLGA MS/HA coating. (**c**) Confocal images of Pac@PLGA MS/HA coating.

The phase composition of HA coating was detected with X-ray diffraction (XRD). XRD analysis showed that Pac@PLGA MS/HA coated Ti had characteristic diffraction peaks of HA at 25.88° and 31.77° (Figure 1a). In small angle X-ray diffraction, there was also a strong peak of octacalcium phosphate (OCP) at 4.77°. These results suggested the existence of HA in the HA coated Ti. Surface morphology observation through electron microscopy confirmed that the surface of the Ti after alkali heat treatment formed a homogeneous, sharp-edged lamellar structure with relatively homogeneous-sized pores in the biomimetic mineralization solution (Figure 1b). The PLGA microspheres electrosprayed on the surface of the biomimetic mineralized Ti were uniformly embedded on the coating surface. Fluorescence double-labeling analysis of the coating showed that the antimicrobial peptides were uniformly distributed on the coating surface with a coating thickness in the range of dozens of microns (Figure 1c).

### 3.2. Release Curve of Pac@PLGA MS/HA Coated Ti

**Figure 2:**
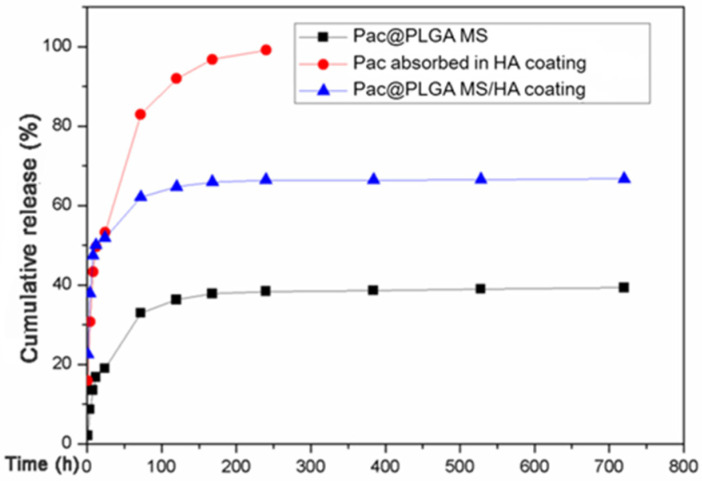
The vitro release of Pac-525 was detected through a cumulative release. Shown are the release curves of Pac-525 in PLGA microspheres, HA coating, and PLGA MS/HA coating.

Analysis of the release of Pac-525 showed that, without PLGA microspheres, the Pac-525 adsorbed in the HA coating was completely released in a short time (more than 80% within 100 h, and completely in 200 h). Further, if Pac-525 was loaded with PLGA microspheres, the Pac-525 was released in a large initial burst followed by a slow release, which could be maintained for over 700 h (Figure 2). The requirements for early burst and long-term slow release were met.

### 3.3. Biocompatibility of Pac-525 and Pac@PLGA MS/HA Coated Ti

Next, we analyzed the proliferation status of cells in different concentrations of Pac-525 (0, 10, 50, 100, 150, and 200 μg/mL). At concentrations of 10 and 50 μg/mL, cell viability was not decreased significantly compared with the control group, but at high concentrations of 100, 150, and 200 μg/mL, the cell viability was slightly weakened. Combined with the antibacterial concentration of Pac-525, the local concentration of Pac-525 in vivo would not induce cell death.

**Figure 3:**
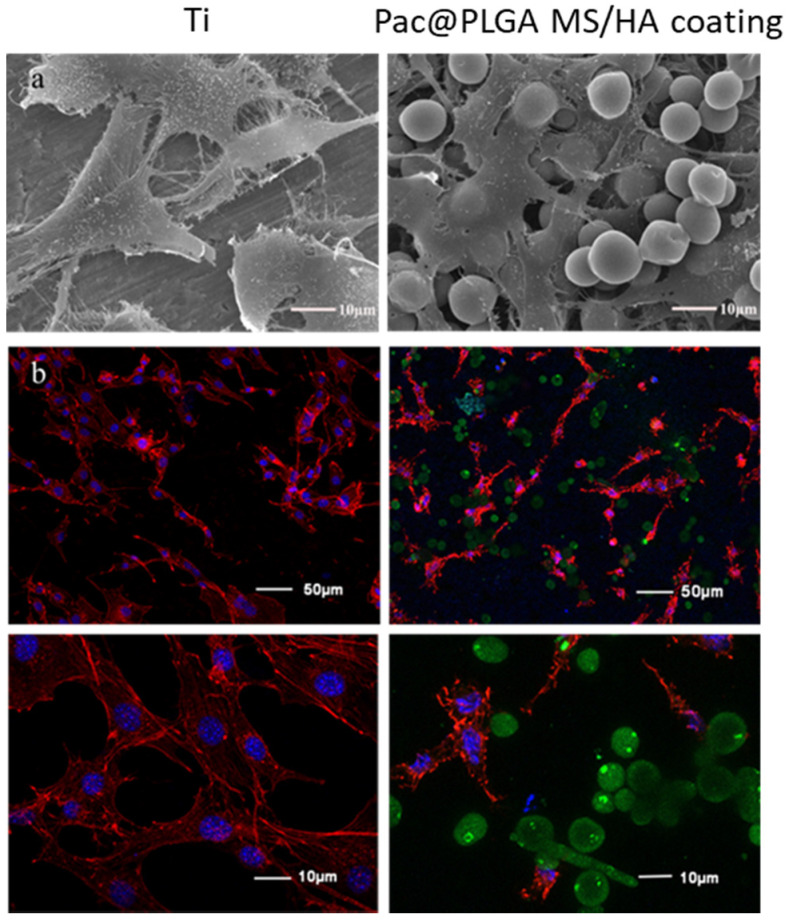
The adhesion of MC3T3-E1 cells cultured on Ti and Pac@MS/HA coating of Ti for 6 h was observed through SEM and CLSM. (**a**) SEM images of MC3T3-E1 cells on Ti and Pac@PLGA MS/HA coated Ti surface. (**b**) Confocal images of MC3T3-E1 cells on Ti and Pac@PLGA MS/HA coated Ti surface. The blue fluorescence is from DAPI in the nucleus; the red fluorescence is from phalloidin in the cell membrane; and the green fluorescence is from Pac@PLGA microspheres.

Next, the biocompatibility of Pac@PLGA MS/HA coated Ti was evaluated. The growth of the osteoblast-like cell line, MC3T3-E1, cultured on the surface of Pac@PLGA MS/HA coated Ti was observed by both SEM (Figure 3a) and CLSM (Figure 3b). After 6 h of cell adhesion, the cells were polygonally stretched on the coating surface with numerous cell pseudopodia, which did not differ significantly from the control group (pure Ti). On basis of the release curve, the Pac-525 released from Pac@PLGA MS/HA coated Ti was not cytotoxic.

**Figure 4:**
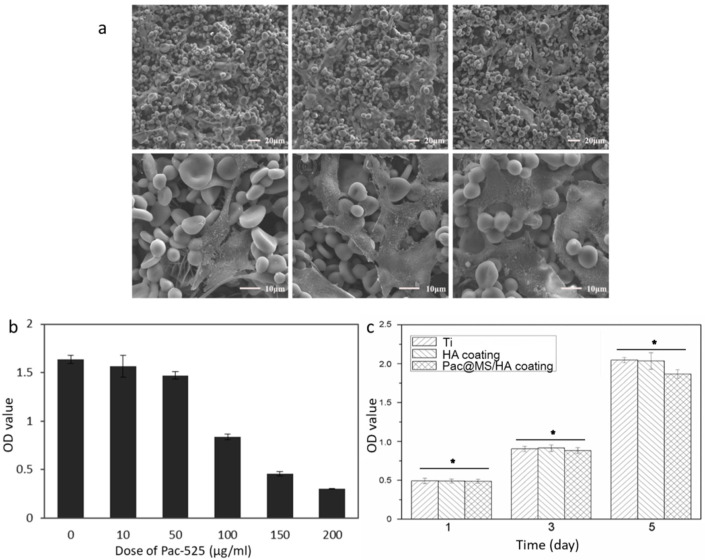
The cytotoxicity and cell proliferation were investigated by CCK-8 and SEM. (**a**) SEM images of MC3T3-E1 cells on Pac@PLGA MS/HA coated Ti for 1, 3, and 5 d. (**b**)The cytotoxicity of Pac-525 on MC-3T3 cells. (**c**) The increased viability of MC3T3-E1 cells on Ti, HA coated Ti, and Pac@PLGA MS/HA coated Ti (* *p* > 0.05).

The cell adhesion was not affected by the coating of Pac@PLGA MS/HA on Ti. Similarly, cell proliferation was also not significantly altered. The results of the CCK-8 assay showed no significant difference between Pac@PLGA MS/HA coated Ti and the control groups (Figure 4a). SEM observation confirmed that the cells proliferated normally on the surface of Pac@PLGA MS/HA coated Ti at 1, 3, and 5 days without any abnormality (Figure 4b).

### 3.4. Antimicrobial Capability of Pac-525 and Pac@PLGA MS/HA Coated Ti

For the MIC experiment, a different dose (0, 1, 2, 4, 8, 16, 32, …) of Pac-525 was used to treat *E. coli* and *S. aureus* at a concentration of 10^5^ CFU/mL for 20 h. The minimum concentration of Pac-525 that kept the medium in clear state would be considered the minimum inhibitory concentration (MIC).

**Figure 5:**
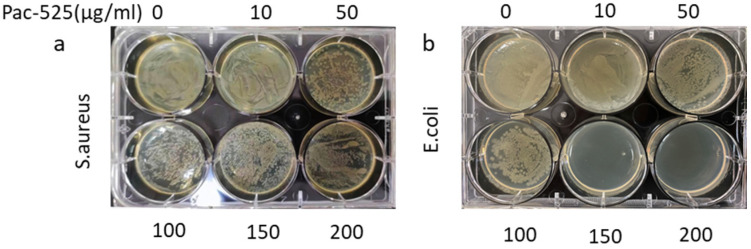
The antibacterial activity of Pac-525 was evaluated with the area of bacteria on agar plate. (**a**) *S. aureus* was inhibited by Pac-525 at the concentration of 50 μg/mL. (**b**) *E. coli* was inhibited by Pac-525 at the concentration of 50 μg/mL.

*E. coli* and *S. aureus* were selected to test the antibacterial effect of Pac-525 and different titanium tablets. Compared with the control group, the results showed that both *S. aureus* and *E. coli* growth area on the agar plates of the groups over 50 μg/mL gradually decreased with the increase of Pac-525 concentration (Figure 5a). Especially for *E. coli*, there were almost no bacteria detected in the 150 and 200 μg/mL groups (Figure 5b).

**Figure 6:**
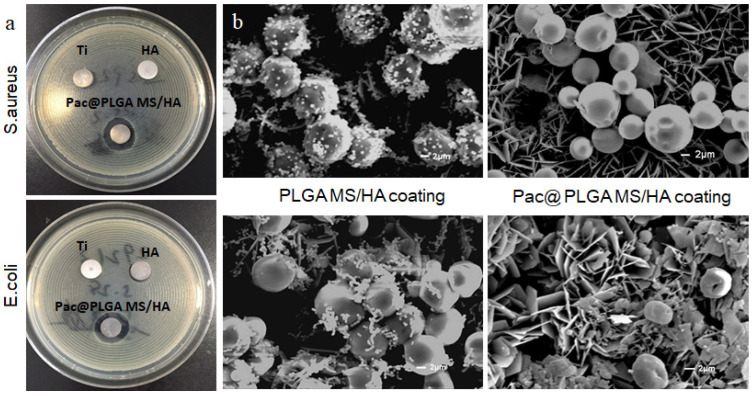
The antibacterial property of Pac@MS/HA coating was detected by bacteriostatic ring measurement and SEM image. (**a**) The bacteriostatic ring diameters of pure Ti, HA coated Ti, and Pac@PLGA MS/HA coated Ti on *E. coli/S. aureus*. (**b**) The comparison of *E. coli/S. aureus* adhering on PLGA MS/HA coated Ti and Pac@PLGA MS/HA coated Ti.

In addition, we selected *E. coli* and *S. aureus* to test the antimicrobial effect of different Ti sheets. The results indicated that only Pac@PLGA MS/HA coated Ti significantly displayed typical morphology of bacteriostatic rings on the agar plate that proved the inhibition of samples on *S. aureus* (1.75 ± 0.052 fold of control) and *E. coli* (1.66 ± 0.079 fold of control). Contrarily, there were almost no bacteriostatic rings observed around the pure Ti and HA samples (Figure 6a). Electron microscopic observation of bacteria on the surface of the Ti revealed almost no detectable bacteria on the surface of the biomimetic mineralized Ti coating with Pac-525, whereas the biomimetic mineralized Ti without Pac-525 had a high level of bacteria (Figure 6b).

## 4. Discussion

The available methods for loading HA on the surface of Ti include plasma spraying, electrophoretic deposition, biomimetic mineralization, and sol–gel methods [5,6]. We dipped the samples into the biomimetic mineralization solution after preparing the biomimetic mineralized Ti with the biomimetic mineralization method [28,29]. Simulated body fluid was our first choice of biomimetic mineralization fluid, but our experience suggested that it not only took a long time to mineralize but also failed to form a uniform coating [30]. Therefore, we tried to use DPBS containing calcium chloride (CaCl_2_) as the biomimetic mineralization fluid. It only took 24 h to form a uniform coating surface. Next, Pac-525 was designed to be adsorbed on the surface to obtain an antibacterial layer. We first tried to use the biomimetic mineralization deposition method, that is, under mild reaction conditions, Pac-525 was deposited on the activated Ti surface. However, it failed to develop an HA coating with Pac-525 evenly combined with the crystal. The possible reason was that the positive charge carried by the antimicrobial peptide affected the binding of calcium ions to the negatively charged alkali heat-treated Ti surface [31,32]. The physical adsorption could achieve an antibacterial HA layer but failed to maintain a long-term activity. Meanwhile, considering the enzyme-induced degradation of antimicrobial peptides by physical adsorption, the electrosprayed PLGA microspheres were used as the drug-release system to load the Pac-525 [26]. The application of PLGA microspheres maintained the local concentration of Pac-525 and protected Pac-525 from being prematurely metabolized (graphical abstract). The crystal of HA grows to be a vertical lamellar structure that left much room and potential for biomimetic recrystallization. PLGA microspheres were inlayed in the pores to avoid dropping from the surface of the implant during the clinical operation.

Although PLGA microspheres displayed advantages as a drug delivery system in previous research, there were also some short comings, such as cytotoxic organic solvent residues, lack of dispersity, and burst release. In our research, these problems were solved by the electrospraying method. The biocompatibility analysis showed that there was no significant difference between samples and pure Ti. The noncytotoxicity of Pac@PLGA MS/HA indicated that there was little organic solvent residue. The electrosprayed PLGA microspheres were also uniformly distributed on the HA layer, thus avoiding accumulation during the simple blend (Figure 1c).

As exogenous materials, orthopedic implants and dental implants cause significant changes in the local environment after placement. In order to maintain the health of the implant, a high concentration of bacteriostatic agents is required to prevent bacterial adherence in the initial postoperative period and subsequently to prevent the development of peri-implantitis, which depends on a long-lasting and effective bacteriostatic environment [1,33]. Therefore, the ideal release profile of the antimicrobial agent is the one consisting of a high initial release rate with a continuous slow release [34,35]. In our study, the ”burst release” of PLGA particles helped to rapidly create a base dose of Pac-525 that exceeded MIC (Supplemental Materials). The antibacterial activity of pure Pac-525 was displayed at the dose of 50 μg/mL (Figure 5a), for which the cell viability was still more than 90% (fold of control), suggesting that Pac-525 was noncytotoxic at this dose of MIC (Figure 4b). Naturally, the distinct bacteriostatic rings on both *E. coli* and *S. aureus* agar plates were observed around the Pac@PLGA MS/HA samples (Figure 6a). Through the SEM, additional microscopic phenomena were displayed to us that, compared to the PLGA MS/HA coating, the application of Pac-525 significantly prevented bacterial adhesion (Figure 6b). The potential of Pac-525 may contribute to its amino acid composition. The rich Trp and Arg could insert into and neutralize LPS, which destroys the integrity of bacterial membrane and eventually leads to its rupture.

In summary, the improved alkali heat treatment shortened the formation time of HA coating. The electrosprayed PLGA microspheres achieved a long-term release of Pac-525 that successfully acquired an antibacterial function.

## 5. Conclusions

In this study, a homogeneous mineralized layer was rapidly deposited on the Ti surface by acid–alkali heat treatment followed by biomimetic mineralization with DPBS containing CaCl_2_. Through physical adsorption and surface electrospraying of Pac-525, the prepared Pac@PLGA MS/HA Ti surface achieved a long-term antibacterial property. The modification of Ti is hoped to prevent the failure of implantation induced by infection.

## Data Availability

The data is available on reasonable request from the corresponding author.

## Supplementary Materials

The following are available online at https://www.mdpi.com/article/10.3390/nano11123306/s1, Supplemenraty Materials: The MIC experiment.
