# Supplementary material for: A Novel Antibacterial Titanium Modification with a Sustained Release of Pac-525
Source: Nanomaterials (Basel). 2021 Dec 6;11(12):3306. doi: 10.3390/nano11123306 (PMC8704243; doi:10.3390/nano11123306)
Supplement: Supplementary file 1 [file nanomaterials-11-03306-s001.zip › nanomaterials-1439920-supplementary.pdf]

Supplemental Materials

For the MIC experiment, different dose (0, 1, 2, 4, 8, 16, 32, …) of Pac-525 was used to treat *E.coli* and *S,aureus* on a concentration of 10^5 CFU/mL for 20 hours. The minimum concentration of Pac-525 that kept the medium in clear state would be thought as the minimum inhibitory concentration (MIC).

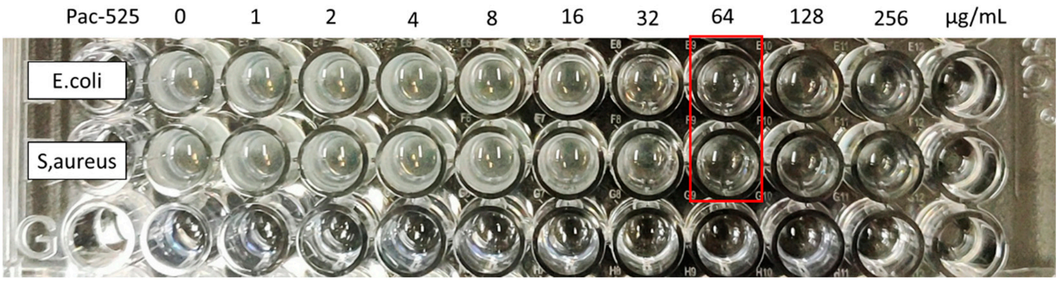

On the dose of 64μg/mL, the medium was clear.
